# Matrine impedes colorectal cancer proliferation and migration by downregulating endoplasmic reticulum lipid raft associated protein 1 expression

**DOI:** 10.1080/21655979.2022.2060777

**Published:** 2022-04-12

**Authors:** Hongtao Ren, Yali Wang, Ya Guo, Mincong Wang, Xiulong Ma, Wen Li, Yuyan Guo, Yiming Li

**Affiliations:** a Department of Radiotherapy, Second Affiliated Hospital of Xi’an Jiaotong University, Xi’an, China; b Department of General Surgery, Second Affiliated Hospital of Xi’an Jiaotong University, Xi’an, China

**Keywords:** Matrine, Erlin1, anti-tumor, proliferation, progression

## Abstract

Matrine exhibits anti-tumor effect on the proliferation and invasion of colorectal cancer (CRC) cells by reducing the activity of the p38 signaling pathway. However, these studies were limited because the underlying mechanism by which matrine inhibited CRC progression remained unclear. In this study, we provided for the first time that endoplasmic reticulum lipid raft associated protein 1 (Erlin1) is a novel target of matrine. Erlin1 was significantly upregulated in tumors and its knockdown suppressed the proliferation and migration of CRC cells, while its overexpression promoted CRC cell growth and migration. Furthermore, Erlin1 overexpression promoted inhibited apoptosis. Importantly, matrine treatment could reverse the oncogenic function of Erlin1 on CRC cell proliferation and migration. When Erlin1 was knocked down, matrine exhibited a more obvious anti-tumor effect in CRC cells. Partly due to this, matrine functions as an important anti-tumor drug and the results discovered here may clarify the mechanisms of matrine application for CRC treatment. CRC patients with low expression of Erlin1 might be more suitable for the treatment of matrine. This study could promote the application of matrine to be a promising therapeutic strategy for CRC patients.

## Graphical abstract

**Figure:**
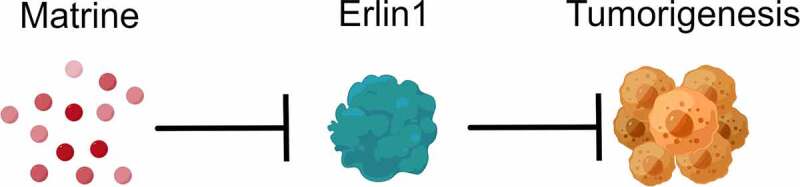

## Introduction

Colorectal cancer (CRC) is the third most common malignancy and the main cause of cancer-related deaths worldwide, with increased global prevalence and a poor prognosis [1]. There are approximately 1,800,000 CRC cases and 800,000 CRC-related death globally [2]. Identifying effective drugs against this malignancy will reduce the health burden in our society. Solid tumor fatalities are largely due to malignant metastasis and therapy resistance. Currently, the combination of a cytotoxic and biological agent is selected as a treatment choice for most CRC patients [3]. Despite the many advances in systemic therapy, nearly 80% of the patients with metastatic CRC die within 5 years of diagnosis [4]. Recently, some novel therapeutic strategies, such as immunotherapy and fungal-derived materials [5,6], are considered promising treatment options for CRC patients. However, once diagnosed at late stage, CRC patients were resistant to the majority of the treatments. Therefore, there is a great need for both clinicians and researchers to seek novel and effective therapeutic approaches to treat CRC.

Increasing evidence has suggested that matrine, a natural alkaloid, and its derivatives originate from the roots and the legume plant of Sophora flavescens (Kushen) and display important roles in multiple biological processes, including anti-tumor, anti-inflammatory, anti-allergic, and antiviral activity [7]. Matrine has been widely used in treating hepatitis, inflammation, nervous system diseases, and cardiovascular disorders [8–12]. Recently, the anti-tumor effects of matrine in several types of solid tumors have been well-documented [13–15]. A previous study suggested that matrine suppresses the capacity of proliferation and invasion of CRC cells by downregulating the p38 signaling pathway [16]. In addition, matrine has been reported to trigger human colon cancer cell apoptosis via the mediation of microRNA-22 [17]. Nevertheless, the exact molecular mechanisms that mediate the biological functions of martine in suppressing CRC progression have yet to be elucidated. Understanding the molecular events during matrine treatment may help the application of matrine for the treatment of CRC patients.

Cancer occurs and develops as a result of disruption of fundamental cellular processes, including cell viability, metastasis, apoptosis, as well as other obvious hallmarks of tumor progression [18]. Metabolic dysregulation, including aerobic glycolysis, lipid, and microbiota-associated amino acid metabolism also play important roles in the development of CRC [19–21]. These disruptions are involved in alterations in the genome and epigenome, such as gene mutation, copy number alterations, epigenetic instability, and chromosome instability, which may depict the potential pathogenesis of CRC development [22,23]. Multiple oncogenes and tumor suppressors are reported to play pivotal roles in the initiation and progression of tumors [24].

The endoplasmic reticulum lipid raft-associated protein (Erlin1), also known as SPFH1, functions as a component of ER membrane proteins, belonging to a family of SFPH domain-containing proteins [25]. Accumulating evidence reveals that Erlin1 could assemble into a complex with Erlin2, playing important roles in ER-related degradation. Indeed, the Erlin1/Erlin2 complex has a clearly defined function in lipid-raft-like domains of the ER [26]. Mutations in Erlin1 may cause slowly progressive early onset amyotrophic lateral sclerosis [27]. Recently, a study suggested that Erlin1 is dysregulated in different tumor differentiation grades, with diagnostic potential for pancreatic adenocarcinoma [28]. However, the role of Erlin1 in CRC remains unclear.

In this study, we hypothesized that matrine might inhibit the growth and migration of CRC cells through the downregulated genes, such as ERLIN1, as checked by microarray screening. We aimed to explore the contribution of the downregulated Erlin1 to CRC growth, migration, and the inhibition effect of matrine on CRC cells.

## Materials and methods

### Cell cultur*e*

Human CRC cells, including HT-29 and RKO, were purchased from the Cell Bank of the Chinese Academy of Sciences (Shanghai, China) and maintained in DMEM/F12 and RPMI 1640 (Corning, Corning, NY, USA), respectively, supplemented with 10% FBS (Gibco, Waltham, MA, USA), 100 U/ml penicillin, and 100 μg/ml streptomycin (Gibco). The above cells were cultured in an incubator at 37°C and 5% CO_2_ [29,30].

### Samples from The Genome Cancer Atlas

In total, 275 colorectal cancer samples and 349 normal samples used in the study were downloaded from The Genome Cancer Atlas (TCGA) data portal (https://portal.gdc.cancer.gov/). The expression of ERLIN1 in these samples was analyzed [31].

### Lentivirus mediated ERLIN1 knockdown and overexpression

Short hairpin RNA-targeting ERLIN1 (shERLIN1; 5’-AGATTATGACAAGACCTTA-3’) and control shRNA (shCtrl; 5’-TTCTCCGAACGTGTCACGT-3’) were synthesized and cloned into the lentivirus expression vector GV115 (Shanghai GeneChem Co., Ltd.). For Erlin1 overexpression, the sequence of ERLIN1 (NM_ NM_006459) was cloned into lentivirus expression vector GV492 (Shanghai GeneChem Co., Ltd.). For lentivirus packaging, the GV115 or GV492 plasmids were co-transfected into 293 T cells with two helper vectors, pHelper1.0 and pHelper2.0, using Lipofectamine® 2000 (Invitrogen; Thermo Fisher, Waltham, MA, USA). After 72 h, the viral supernatants were collected by ultracentrifugation. Following transfection with lentiviruses for 48 h (MOI = 10), the transduced cells were selected with 5 µg/ml puromycin (Sigma-Aldrich, St. Louis, MO, USA) [32].

### Microarray screening

HT-29 cells were treated with varying doses of matrine to determine the IC50 [33]. After treatment with 1.722 mg/ml matrine for 48 h, total RNA was extracted using TRIzol reagent (Thermo Fisher), followed by microarray detection. The microarray analysis was performed by Shanghai GeneChem Co., Ltd. The dysregulated genes were identified based on statistical significance at *P* < 0.05 and fold-change > 1.5.

### MTT assay

The capacity for cellular proliferation was evaluated using the MTT assay with the Cell Proliferation Reagent Kit I (MTT) (Roche) [33]. HT-29, DLD-1, and RKO cells (2 × 10^3^ cells/well) were seeded into a 96-well plate for 24 h. Then, MTT solution, diluted in fresh medium, was added to the plate for 2 h. Subsequently, the medium was replaced with DMSO at 37°C for 15 min, after which the absorbance was measured at 490 nm.

### HCS assay

Cell viability was measured by high-content screening (HCS). Briefly, after varying treatments, cells were seeded in 96-well plates for 24 h (2 × 10^3^ cells/well). A Celigo image cytometer system (Nexcelom Bioscience LLC, Lawrence, MA, USA) was used to capture and determine the number of cells daily. The green cells were successfully infected, and the number of cells was calculated using the Celigo image cytometer system (Nexcelom) [34].

### Apoptosis assay

For apoptosis detection, cells from different treatment groups were obtained and fixed in 70% ice-cold ethanol (diluted with ddH_2_O) at 4°C overnight. The cells were then stained with Annexin V/APV kit (Yeasen, Shanghai) according to the manufacturer’s instructions, followed by detection with a flow cytometer (Millipore, Guava easyCyte HT).

### Cell cycle assay

CRC cells (1 × 10^5^/well) were seeded in 6-well plates for 24 h and were collected after centrifugation at 700 rpm for 5 min. Subsequently, the cells were washed twice with PBS and fixed in 70% ethanol for 12 h. After staining with 0.02% propidium iodide (PI) for 30 min at 4°C, cell cycle distribution was determined using A flow cytometer (Millipore, Guava easyCyte HT) [35].

### Transwell assay

The cell migration capacity was examined using a transwell migration assay. The cells were seeded into the upper chamber and maintained in RPMI 1640 medium without FBS. The lower chamber was then supplemented with RPMI 1640 medium with 10% FBS. After incubation at 37°C for 48 h, the invasive cells were fixed with 4% paraformaldehyde, stained with 0.1% crystal violet solution (Beyotime, China), and observed under a microscope (XD, Ningbo Sunny Instruments CO., LTD.) [36].

### Western blot analysis

Protein expression levels were analyzed using western blot analysis. Briefly, total protein was harvested from the cells using RIPA lysis buffer (Beyotime, China), and protein concentrations were detected using a BCA kit (Thermo). After SDS-PAGE, the following primary antibodies against the target proteins were used: Erlin1 (1:1000, ab171372; Abcam), GAPDH (1:10,000, SC-32233; Santa Cruz Biotechnology), and β-actin (1:5000, SC-69879; Santa Cruz Biotechnology). GAPDH or β-actin was used as an endogenous control to normalize target protein expression [37].

### Real-time quantitative (qRT-PCR) assay

Total RNA from cells was extracted using TRIzol (Invitrogen), and 1 μg of cDNA was synthesized using the Prime Script RT reagent Kit (Takara). The qPCR assay was performed using the SYBR Green PCR kit. The following primers in the present study were used: ERLIN1, F: 5’- AATATGCCACCTCAAACAAGCA-3’, R: 5’- GGGTTCAAGAGCCTCCTTAGAG-3’; GAPDH, F: 5’- GGGTTCAAGAGCCTCCTTAGAG-3’, R: 5’-CACCCTGTTGCTGTAGCCAAA-3’. qPCR and data analysis were conducted on an MX3000p (Agilent), and the mRNA expression levels were normalized to that of GAPDH [38].

### Statistical analysis

Statistical analysis was performed using SPSS 15.0 (SPSS Inc., Chicago, IL, USA). All values are presented as the mean ± SD. The unpaired Student’s t-test, one-way ANOVA, and Tukey’s post-hoc test were used to analyze the differences between the two groups. *P* < 0.05 was considered statistically significant.

## Results

We suspected that martine suppressed the proliferation and migration of CRC cells through downregulating Erlin1. Therefore, this study intended to investigate the regulation of martine on Erlin1, the role of Erlin1 on CRC cell proliferation and migration treated with or without martine. We examined the dysregulated genes in CRC cells after martine treatment. Gain-of-function and loss-of-function assays were performed to study the role of Erlin1 overexpression and knockdown on CRC cell apoptosis, cell cycle, proliferation, and migration, which were analyzed by flow cytometry, MTT, and transwell. qRT-PCR and Western blot were carried out to examine mRNA and protein expression, respectively.

### Martine downregulated Erlin1 in CRC cells

**Figure 1.**
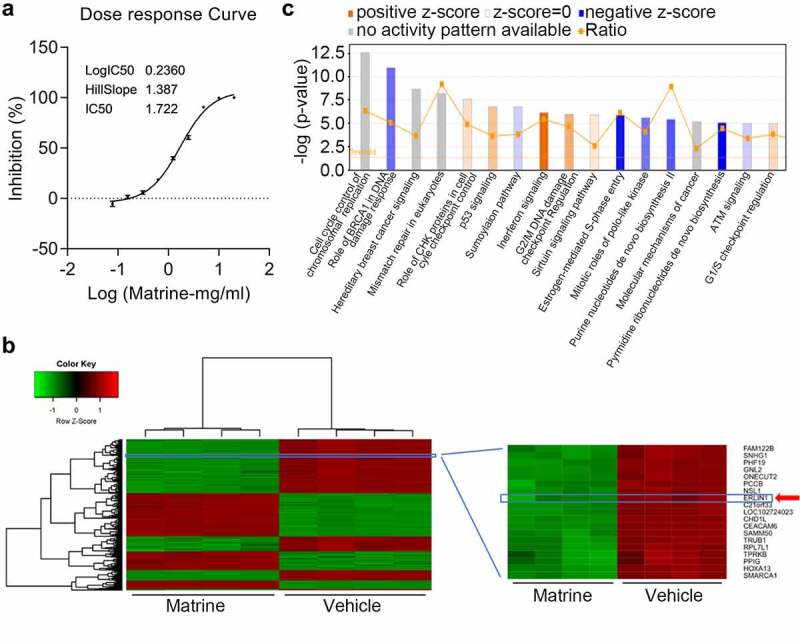
Martine downregulated Erlin1 in CRC cells.

Increasing evidence suggests that martine plays a pivotal role in suppressing CRC progression. To clearly investigate the anti-tumor roles of matrine, the IC50 was determined under different doses of matrine in HT-29 cells. Based on this, the cells were treated with 1.722 mg/ml matrine for 24 h, followed by microarray analysis to identify differentially expressed genes contributing to matrine-mediated tumor inhibition (Figure 1a). As shown in Figure 1b, systematic variations (≥ two-fold, *P* < 0.05) were screened for mRNA expression levels and visualized by hierarchical clustering. Among the significantly expressed genes, Erlin1 was significantly downregulated following matrine treatment. The IPA analysis revealed that these dysregulated genes were typically involved in the role of BRCA1 in the DNA damage response, interferon signaling, pyrimidine ribonucleotide de novo biosynthesis, and cell cycle (Figure 1c).

### Erlin1 was strongly upregulated in CRC cells

**Figure 2.**
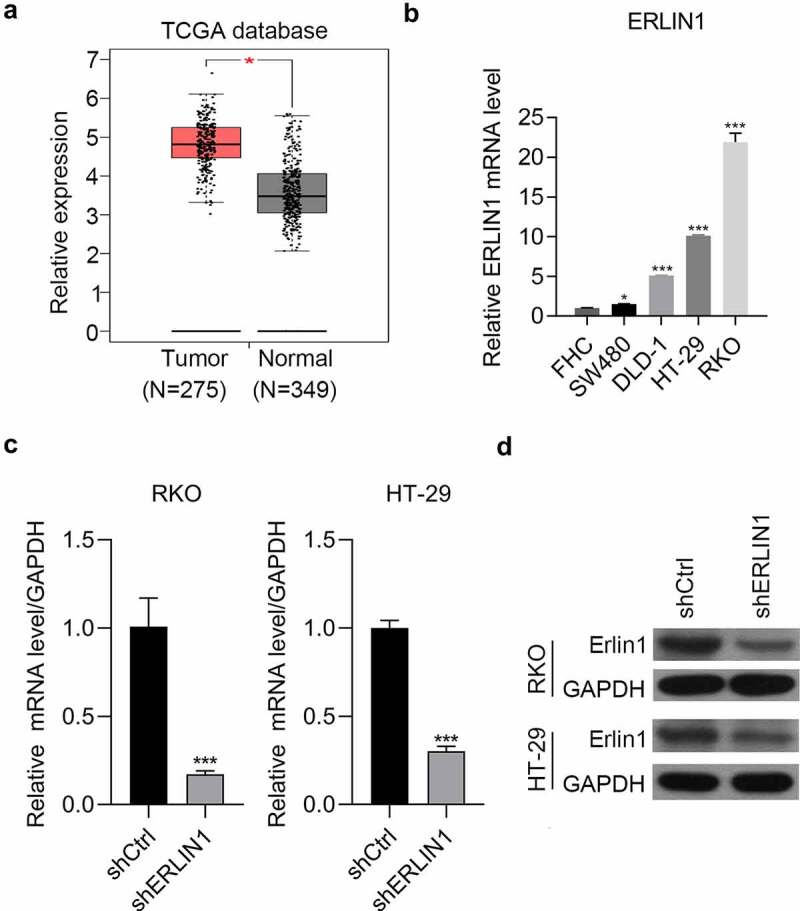
Erlin1 was strongly upregulated in CRC tissues.

Erlin1 was suppressed by the matrine treatment in HT-29 cells and was detected in CRC tissues by analyzing The Cancer Genome Atlas database, which revealed that it was significantly upregulated compared with adjacent tissues (Figure 2a). Additionally, Erlin1 was detected in the normal colorectal cell line FHC and in four CRC cell lines by qRT-PCR. Compared with FHC, CRC cells had higher expression of Erlin1. In addition, the expression of Erlin1 was relatively higher in RKO and HT-29 cells than that in DLD-1 and SW480 cells (Figure 2b). The KD efficiency mediated by shRNA technology was determined by qRT-PCR and western blot analysis, which indicated that the expression of Erlin1 was significantly knocked down using shRNA technology in HT-29 and RKO cells (Figures 2C and 2d).

### Downregulation of Erlin1 suppressed CRC progression

**Figure 3.**
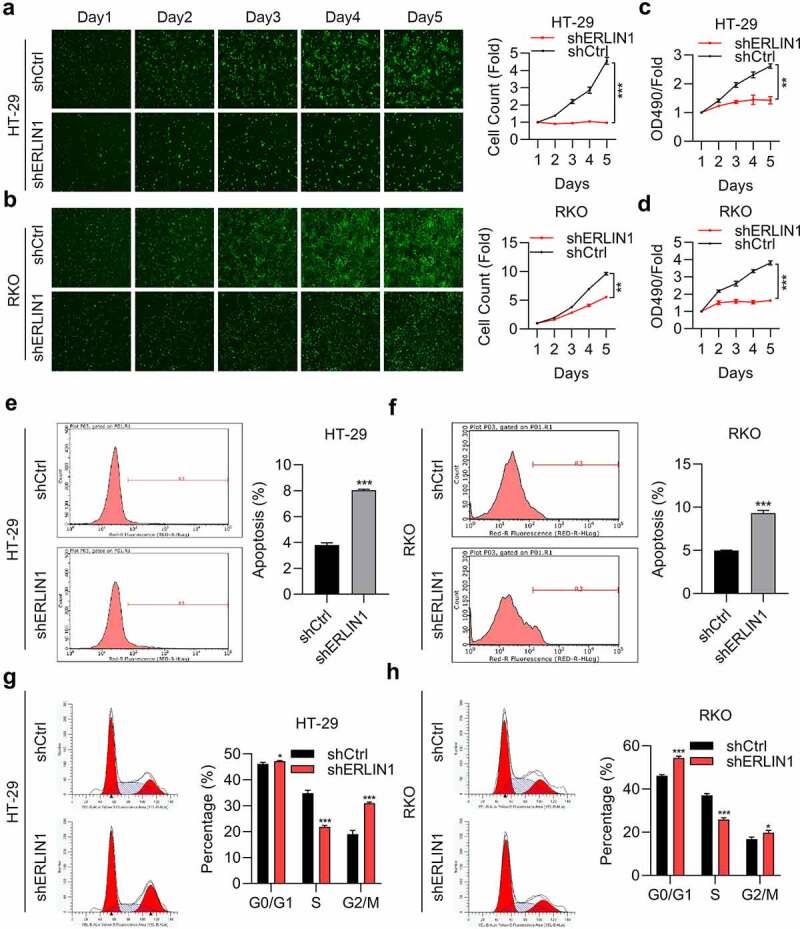
Erlin1 knockdown suppressed CRC progression.

To investigate the role of Erlin1 in CRC development, the capacity of cell proliferation was determined by MTT and HCS assays. As shown in Figures 3A to 3d, cell proliferation was suppressed after Erlin1 KD. We then determined whether Erlin1 influenced CRC cell apoptosis, and the flow cytometry results indicated that the rate of apoptosis was enhanced in Erlin1 KD cells (Figures 3E and 3f). We next tested the effect of Erlin1 expression on the cell cycle, which suggested that Erlin1 silencing triggered the arrest of CRC cells in the G1 and G2/M phases, and decreased the number of cells in the S phase in both HR-29 and RKO cells (Figures 3G and 3h).

### Overexpression of Erlin1 promoted CRC proliferation and migration which can be reduced by matrine

**Figure 4.**
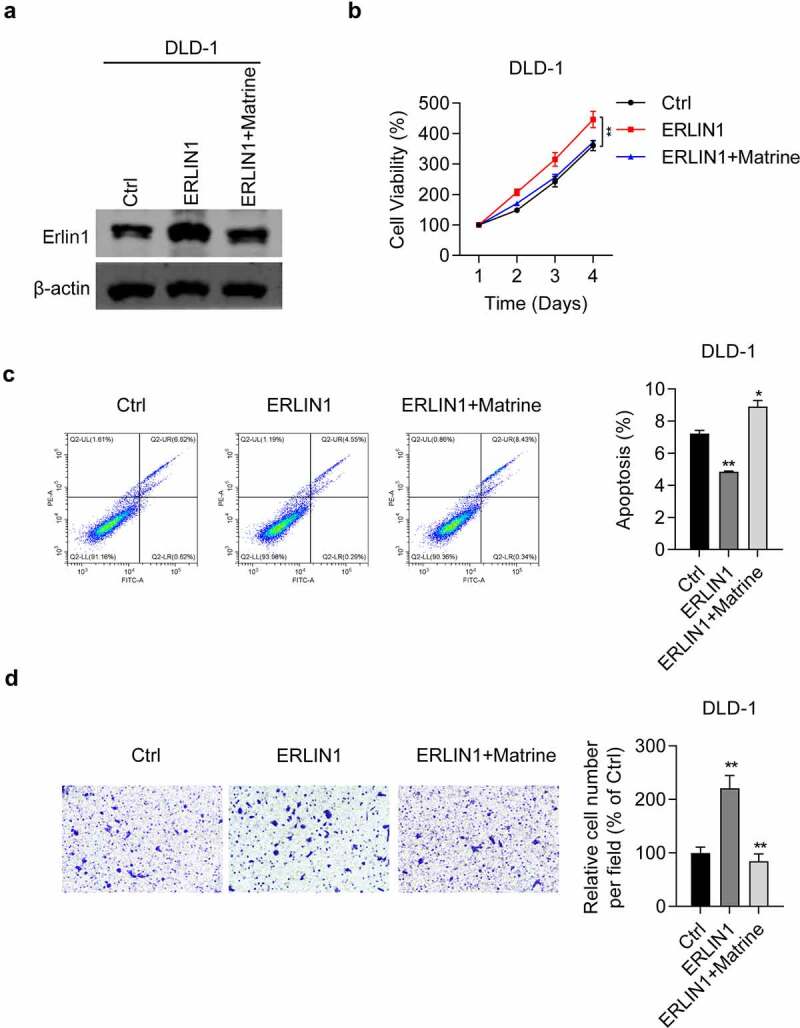
Overexpression of Erlin1 promoted CRC proliferation and migration which can be reduced by matrine.

Next, we examined the effect of Erlin1 overexpression on CRC progression. First, we determined the efficiency of Erlin1 overexpression. As shown in Figure 4a, Erlin 1 was significantly overexpressed in DLD-1 cells, whereas matrine treatment downregulated Erlin1. The MTT assay showed that when Erlin1 overexpression promoted DLD-1 cell viability, matrine treatment reversed the effect (Figure 4b). Furthermore, apoptosis analysis revealed that elevated Erlin1 levels inhibited apoptosis in DLD-1 cells, which could be enhanced by matrine treatment (Figure 4c). Importantly, we also showed that migratory ability was significantly enhanced by Erlin1 overexpression in DLD-1 cells (Figure 4d). Consistently, matrine treatment reversed the oncogenic role of Erlin1 on the migration of DLD-1 cells (Figure 4d). Taken together, these results demonstrate the oncogenic potential of Erlin1 in CRC.

### Matrine inhibited CRC cell progression by downregulating Erlin1

**Figure 5.**
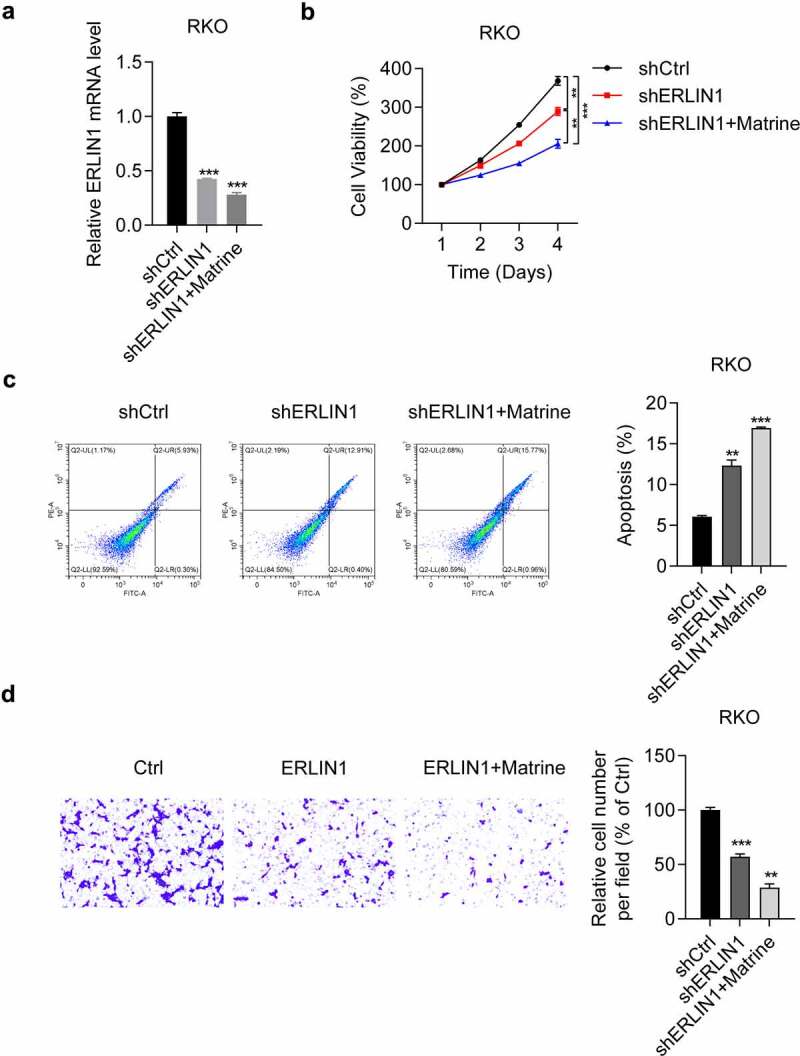
Erlin1 knockdown enhanced anti-tumor effects of matrine in CRC cells.

Subsequently, we determined the role of Erlin1 in the suppression of matrine-induced CRC cell progression. In RKO cells, we observed that both sh-ERLIN1 and sh-ERLIN1 combined with matrine inhibited Erlin1 expression, as detected by qRT-PCR (Figure 5a). We further examined the effects of Erlin1 knockdown on matrine-treated RKO cells. The results suggested that the anti-tumor effect of matrine, including inhibition of cell proliferation (Figure 5b), induction of cell apoptosis (Figure 5c), and suppression of cell migration (Figure 5d), could be strengthened by both matrine treatment and Erlin1 suppression. Collectively, these results suggest that matrine exerts its anti-tumor effect on CRC progression by decreasing Erlin1 expression.

## Discussion

CRC remains a life-threatening malignancy, even though there are great improvements in therapies. Approximately 6–10% of CRC patients are caused by genetic mutations, including TP53 and WNT/β-catenin signaling [39]. For these patients, targeted therapies are developed and some of the patients benefit from the small molecules targeting WNT/β-catenin signaling [40]. However, the molecular drivers of most CRC patients are unclear and effective drugs are limited for the treatment of these patients. Thus, novel molecular targeted treatments are needed. Matrine, a natural herb widely applied in China, has been shown to exert anti-tumor activities in multiple cancers, including gastric and cervical cancers [41,42]. However, the exact mechanism by which matrine impedes CRC proliferation and migration has not been elucidated. In the present study, we demonstrated that matrine exhibits potent anti-tumor activity by downregulating Erlin1 expression in CRC cells. As a traditional anti-tumor drug, matrine suppresses the proliferation and migration of CRC cells, arrests cell cycle progression at the G0/G1 phase, and induces apoptosis. The underlying mechanisms are associated with several tumor-related genes. Thus, our data reveals that matrine could be considered a novel therapeutic treatment for CRC.

Growing research has revealed that matrine treatment exerts its anti-cancer effect by regulating specific genes in multiple solid tumors [43,44]. However, the precise mechanisms underlying the anti-cancer activity of matrine against CRC cells remain unclear. As a previous study suggested, modified TIM2 could enhance the effects of tumor suppression in hepatocarcinoma cells treated with matrine [44]. In addition, another study showed that matrine exerted its anti-tumor effects on dendritic cells via regulating TLR pathways [45]. Except for protein coding genes, it has also been found that matrine induced apoptosis and inhibited cell proliferation in thyroid cancer cells by suppressing miR-182-5p expression [13]. With this, we treated CRC HT-29 cells with matrine, followed by RNA microarray analysis. We showed that thousands of genes were upregulated and downregulated after matrine treatment. Some signaling, such as cell cycle and BRCA1 related, are significantly regulated by matrine. These results suggest that matrine exhibited its anti-cancer effect depending on the dysregulation of the genes as listed in the microarray data. Therefore, understanding the contribution of the dysregulated genes to matrine could help the precise application of matrien for CRC. Among the differentially expressed genes, Erlin1 was significantly downregulated upon matrine treatment in HT-29 cells, which may play important roles in matrine-mediated anti-tumor effects.

Notably, Erlin1 is an ER membrane-related protein with an N-terminal transmembrane domain. Previous studies found that Erlin1 could form a complex with Erlin2, which could play an important role in cholesterol binding, lipid metabolism regulation, and influencing liver fat deposition [46,47]. Both Erlin1 and Erlin2 play pivotal roles in controlling cell fate by regulating lipid metabolism [48]. Another study demonstrated that Erlin1 interacting with AMBRA1 at mitochondria-associated membranes raft-like microdomains is essential for autophagosome formation [49]. Erlin1 is also correlated with hepatitis C virus infection and may have the potential to be targeted for the development of vaccine, such as hepatitis C virus vaccine and influenza vaccine [50,51]. Despite these numbers of reports have illustrated the role of Erlin1 cell fate, metabolism or virus infection, limited studies are aimed to explore the role of Erlin1 in cancer development. In addition, the potential function of Erlin1 in CRC progression and development remains unclear. In this study, we first investigated the potential role in CRC development and found that matrine induced Erlin1 upregulation in CRC cells. We then determined the effects of Erlin1 expression on CRC progression, which revealed that Erlin1 knockdown effectively suppressed CRC proliferation and migration, blocked the cell cycle at the G1 phase, and induced apoptosis. In contrast, overexpression of Erlin1 promoted cell progression in CRC cells. Collectively, these results indicate that Erlin1 expression affects CRC progression. Next, we determined whether matrine inhibits CRC development and progression by regulating Erlin1 expression. The results suggested that Erlin1 knockdown enhanced the anti-tumor effects of matrine against CRC cells, which indicates that matrine suppresses CRC progression by regulating Erlin1 expression. We further verified that matrine suppresses tumor development.

### Conclusion

Taken together, the present study demonstrates that matrine therapy inhibits the development and progression of CRC cells by controlling cell proliferation, apoptosis, cell migration, and the cell cycle by downregulating Erlin1 expression. This study illustrated not only the oncogenic role of Erlin1 in CRC but also the contribution of Erlin1 to matrine’s anti-cancer effect. Since there are no studies reporting the role of Erlin1 on cancer, our study will be helpful for the research studies focusing on matrine, CRC diagnosis, or other CRC drugs. As a traditional agent, matrine has the potential to be developed into a specific targeting agent against CRC.
